# SleepStageNet: A Lightweight and Explainable Deep Learning Architecture for Multi-Channel Sleep Staging

**DOI:** 10.3390/brainsci16080860

**Published:** 2026-08-14

**Authors:** Ali Alhazmi

**Affiliations:** 1 Department of Computer Science, College of Engineering and Computer Science, Jazan University, Jazan 45142, Saudi Arabia; alihazmi@jazanu.edu.sa; 2 Engineering and Technology Research Center, Jazan University, Jazan 82817, Saudi Arabia

**Keywords:** sleep stage classification, polysomnography, deep learning, convolutional neural network, gated recurrent unit, self-attention, explainability, Indian sleep dataset, stroke patients, cross-dataset validation, ISRUC-SLEEP

## Abstract

**Background/Objectives:** Automatic sleep staging from polysomnography (PSG) is particularly challenging in clinical cohorts with neurological disorders. **Methods:** This study presents SleepStageNet, a compact model (1.075M parameters) that integrates a dual-branch convolutional epoch encoder, feature gating, a bidirectional gated recurrent unit, and multi-head self-attention for five-class staging from five PSG channels (C3, C4, EOG1, EOG2, and chin EMG). The individual operations are adapted from established architectures; the study contribution is their compact integration and controlled evaluation in an Indian acute stroke cohort. **Results:** Of the 100 recordings in the Indian Sleep Polysomnography (iSLEEPS) resource, 95 satisfied the five-channel extraction criteria, yielding 78,323 annotated epochs. Subject-independent 10-fold stratified group cross-validation produced an accuracy of 73.91 ± 2.24%, a macro F1-score of 67.29 ± 1.96%, and a Cohen’s κ of 0.634±0.029 (sample standard deviations). A matched single-branch encoder obtained κ=0.635 (full minus single branch: Δκ=−0.001, Holm-adjusted p=0.846), while matched C4-only input obtained κ=0.600 (full minus C4-only: Δκ=0.033, Holm-adjusted p=0.008). Grad-CAM and temporal attention visualizations provided qualitative evidence of physiologically plausible focus, while channel occlusion quantified the contribution of each signal. Without fine-tuning, a 10-model ensemble obtained κ=0.614 on ISRUC-SLEEP Subgroup III (10 healthy subjects; 8889 epochs). **Conclusions:** These results establish a reproducible reference for this clinical cohort while identifying the need for broader external and prospective validation.

## 1. Introduction

Sleep supports cognition, memory consolidation, immune regulation, and metabolic homeostasis. Obstructive sleep apnea (OSA) alone is estimated to affect approximately 936 million adults globally [1]. The burden of sleep disorders is also high in India: systematic reviews report adult prevalence estimates of 25.7% for insomnia, 37.4% for OSA, and 10.6% for restless legs syndrome [2]. Relative to this burden, sleep research and clinical infrastructure in the Indian subcontinent remain limited compared with those in Western countries.

Polysomnography (PSG) is the reference standard for assessing sleep architecture. It records multiple physiological signals, including electroencephalography (EEG), electrooculography (EOG), and electromyography (EMG). Following American Academy of Sleep Medicine (AASM) criteria [3], trained technologists assign each 30 s epoch to Wake (W), non-REM Stage 1 (N1), non-REM Stage 2 (N2), non-REM Stage 3 (N3), or rapid eye movement (REM) sleep. Manual scoring can require 2–4 h per recording and shows inter-scorer agreement of around κ=0.72–0.82 [4], making it a bottleneck in clinical sleep research.

Deep learning has advanced automatic sleep staging. DeepSleepNet [5], for example, combines convolutional feature extraction with bidirectional LSTM sequence modeling for raw EEG. Subsequent work has used attention mechanisms [6], hierarchical recurrent networks [7], and transformers [8]. Important limitations remain. Stage distributions are imbalanced, commonly favoring N2 over N1 and REM, which can bias predictions toward prevalent classes. Much of the literature also focuses on healthy Western cohorts such as SHHS [9] and Sleep-EDF [10], leaving uncertainty for other geographic and clinical populations. Model opacity further limits clinical interpretation. These issues are particularly relevant in neurological cohorts, in which fragmented sleep, reduced REM, and increased N1 can alter sleep architecture [11,12].

This paper presents SleepStageNet, a compact deep learning model evaluated on the Indian Sleep Polysomnography (iSLEEPS) resource, which contains 100 PSG recordings from acute stroke patients. SleepStageNet combines multi-scale convolutional feature extraction, feature gating, bidirectional recurrent sequence modeling, and self-attention to represent epoch-level signals and inter-epoch context. These operations were established in prior work; the methodological contribution evaluated here is a parameter-efficient integration for five-channel clinical PSG, together with matched input and encoder comparisons, complementary explanation analyses, and zero-shot external evaluation.

The contributions of this paper are the following:

- SleepStageNet integrates a dual-branch CNN, feature gating, a BiGRU, and self-attention in a 1.075M-parameter model for five-channel PSG sleep staging.
- A subject-independent 10-fold reference evaluation is established on the 95 iSLEEPS recordings that satisfy the five-channel extraction criteria.
- Matched full-protocol comparisons evaluate the dual-branch encoder and five-channel input against a single-branch encoder and C4-only input; a separate pilot ablation provides exploratory component-level estimates.
- Grad-CAM, temporal attention visualization, and channel occlusion characterize the model’s learned focus without treating qualitative saliency as proof of a physiological mechanism.
- Zero-shot evaluation on ISRUC-SLEEP Subgroup III [13] measures transfer from Indian stroke recordings to healthy Portuguese recordings acquired with a different system and montage.

The rest of this paper is structured as follows: Section 2 discusses related work on sleep staging automation. Section 3 details the dataset, the preprocessing pipeline, the proposed architecture, and the training strategy. Section 4 presents the results of the experiments, the ablation study, the explainability analyses, and external validation. Section 5 discusses the findings and their implications. Finally, Section 6 concludes the paper and proposes avenues for future research.

## 2. Related Work

End-to-end deep learning has shifted automatic sleep staging away from classical methods that require manually engineered features. This section reviews the most relevant approaches by methodology.

Earlier systems combined time, frequency, and time–frequency domain features with classical classifiers. Lajnef et al. [14] reported approximately 84% accuracy on a 15-subject dataset using wavelet and spectral features with multiclass support vector and decision tree classifiers. Such pipelines require domain knowledge for feature selection, motivating representation learning approaches.

Convolutional networks enable representation learning directly from raw EEG. Supratak et al. [5] developed DeepSleepNet with large- and small-filter CNN branches followed by bidirectional LSTM. On Sleep-EDF-20, it achieved 82% accuracy and a 76.9% macro F1-score. Chambon et al. [15] compared single- and multi-channel configurations on MASS and reported up to 87% accuracy with a multivariate, multimodal CNN using EEG, EOG, and EMG.

Having recognized that sleep stage changes can be temporally predictable (e.g., a Wake-to-REM transition can only occur after some NREM periods), a number of studies attempted to use recurrent neural networks for modeling the sleep stages. Phan et al. [7], for example, devised SeqSleepNet, which is a hierarchical end-to-end recurrent neural network model at both the epoch level and the sequence level (using attention for intra-epoch modeling and bidirectional LSTM for inter-epoch modeling). The sequence-to-sequence architecture of SeqSleepNet enables it to produce hypnograms that are smooth, and it achieved an accuracy of 87.1% on the Sleep-EDF-20 dataset. Seo et al. [16] developed IITNet, which achieved competitive performance on the Sleep-EDF dataset through the use of intra-epoch and inter-epoch temporal modeling via sub-epoch feature extraction.

Attention mechanisms provide an additional approach to temporal modeling. AttnSleep [6] combines adaptive feature recalibration, temporal context encoding, and multi-head attention, achieving reported N1 F1-scores of approximately 40–48% across benchmarks. SleepTransformer [8] uses a transformer architecture and achieved a reported 85.0% accuracy on Sleep-EDF-20. Attention weights can be inspected, but they should not be equated with a causal explanation.

Alongside these architecture-based works, Perslev et al. [17] proposed U-Sleep, an architecture designed to be resilient to cross-dataset generalization and to pretrain on multiple datasets. After training on more than 15,000 PSG recordings from a variety of sources, U-Sleep exhibited a strong performance across multiple recording configurations. This demonstrated that large-scale pretraining benefits sleep staging models. Guillot and Thorey [18] introduced RobustSleepNet, which uses domain-adaptive batch normalization for cross-dataset transfer learning and achieved a strong performance in cross-dataset transfer learning with little fine-tuning.

Automated sleep staging comprises an extensive body of work, yet most of the studies undertaken have been on relatively healthy subjects or general sleep clinic patients, while studies on sleep staging in clinical populations, especially those with neurological disorders, are scarce. Brown et al. [12] pointed out the complications of predicting sleep-disordered breathing in post-stroke patients, a situation where the sleep architecture is changed and both manual and automated scoring are complicated. This study helps fill the gap in the literature relating to various clinical populations by analyzing the iSLEEPS dataset, which contains PSG recordings from patients after acute stroke.

The iSLEEPS dataset has recently been formalized as a clinical PSG benchmark for ischemic stroke patients [19]. Bose et al. [20] evaluated SE-ResNet plus bidirectional LSTM using single-channel C4:M1 EEG and 10-fold cross-validation, reporting 74.7% accuracy, a 67.7% macro F1-score, and κ=0.64. These point estimates are slightly higher than the five-channel SleepStageNet estimates reported in this study, but the preprocessing, temporal window, architecture, and fold membership are not identical. Bose et al. also documented a generalization gap when models trained on healthy cohorts were applied to iSLEEPS. This prior result motivated both a matched C4-only experiment within the present pipeline and cautious framing of the five-channel contribution.

Deep learning methods have achieved strong results on Sleep-EDF, MASS, and SHHS, but evidence from neurological cohorts and non-Western populations remains limited. The present study does not claim novelty for its CNN, gating, recurrent, or attention operations individually; instead, it evaluates whether their compact integration can provide a useful five-channel reference for iSLEEPS, whether the additional channels and dual-branch encoder improve performance under matched conditions, what information the trained model uses, and how well the resulting models transfer without fine-tuning to ISRUC-SLEEP Subgroup III.

## 3. Materials and Methods

This section describes the datasets used for training and external validation, the preprocessing pipeline for the raw PSG signals, the architecture of SleepStageNet and its components, and the training and evaluation methodologies.

### 3.1. Dataset Description

The Indian Sleep Polysomnography (iSLEEPS) resource, publicly released through Zenodo and the Data Foundation/IHub-Data portal [19,21], contains full-night PSG recordings from 100 subjects (77 male, 23 female; ages 22–78 years; mean 50.5±12.0 years; BMIs 15.1–106.0 kg/m^2^; mean 28.0±13.1 kg/m^2^). All subjects are acute stroke patients who underwent clinical PSG. The cohort includes middle cerebral artery territory, basal ganglia, and cerebellar infarcts, and many subjects have comorbid obstructive sleep apnea (AHI range 0.0–81.6; mean 25.2; median 20.75).

Recordings were acquired with a SOMNOmedics PSG system (SOMNOmedics AG, Randersacker, Germany), stored in European Data Format, and sampled natively at 256 Hz. Each recording contains 22–28 signals, including EEG (C3:M2, C4:M1, O1:M2, O2:M1), EOG (E1:M2, E2:M2), chin EMG, ECG, respiratory signals (airflow, thoracic and abdominal effort, and SpO_2_), position, and actigraphy. A trained technologist annotated 30 s epochs as W, N1, N2, N3, or REM according to AASM criteria [3]. Epochs labeled as ‘artifact’ or ‘movement’ were excluded.

**Figure 1:**
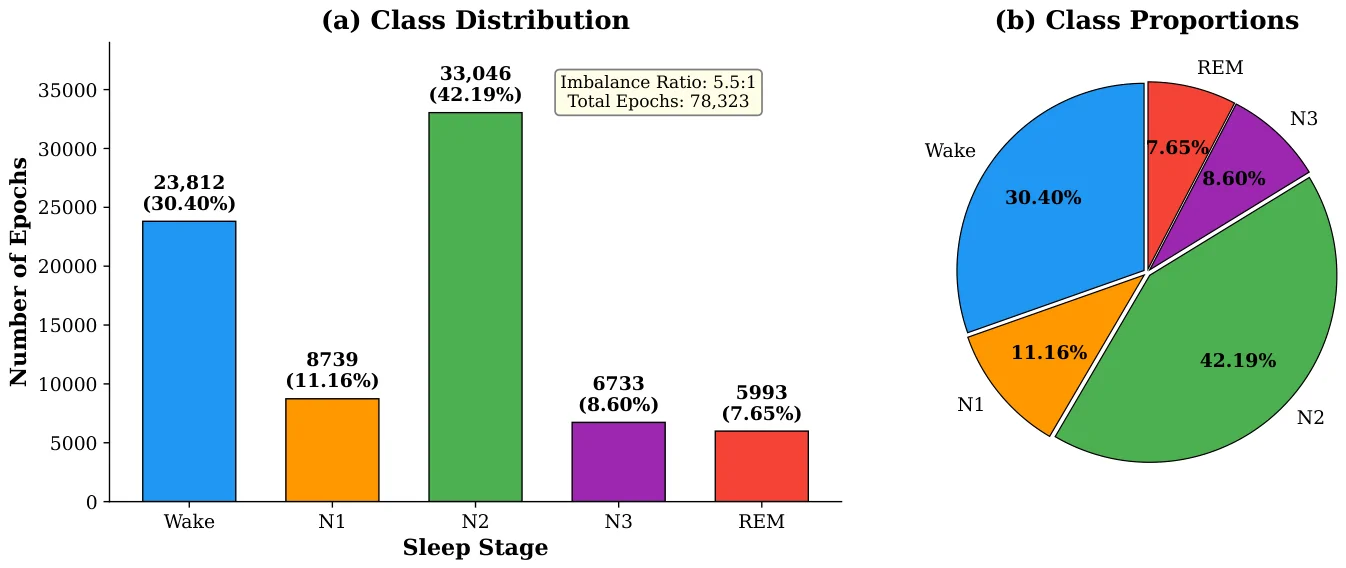
Class distribution of the iSLEEPS dataset. (**a**) Bar chart showing the number of epochs per sleep stage with corresponding percentages: N2 is the most prevalent class (42.19%), while REM is the least prevalent (7.65%), yielding an imbalance ratio of 5.5:1. (**b**) Pie chart showing the proportional distribution of sleep stages.

Five channels used in routine sleep staging were selected: two central EEG channels (C3 and C4), two EOG channels (EOG1 and EOG2), and one chin EMG channel. Five recordings did not satisfy the common five-channel extraction and montage-mapping criteria and were excluded before model development. Consequently, the analyzed set comprises 95 subjects and 78,323 epochs. Table 1 distinguishes the source cohort from the analyzed set, and Figure 1 shows the class imbalance in the analyzed epochs. Table 2 summarizes the corresponding stage counts and proportions.

Subgroup III [13] of the ISRUC-SLEEP dataset serves as an external independent validation dataset to evaluate the cross-dataset generalizability of the proposed model. This subgroup consists of full-night PSG recordings of 10 healthy subjects who do not have any known sleep or neurological disorders and were recorded at the Sleep Medicine Centre of the Hospital of Coimbra University, Portugal. Each recording was scored by two certified sleep specialists according to AASM guidelines, and the annotations of the first scorer were selected for this evaluation.

The ISRUC recordings were collected at a native sampling rate of 200 Hz with 19 channels, including EEG (utilizing the 10–20 system), EOG (LOC-A2, ROC-A1), and chin EMG (X1). Five channels corresponding to those used in the iSLEEPS pipeline are extracted: C3-A2 (mapped to C3), C4-A1 (mapped to C4), LOC-A2 (mapped to EOG1), ROC-A1 (mapped to EOG2), and X1 (mapped to EMG). Across the 10 subjects, there are 8,889 annotated 30 s epochs. Sleep stages in ISRUC are labeled as 0 (Wake), 1 (N1), 2 (N2), 3 (N3), and 5 (REM), which are mapped to the standard 5-class scheme consistent with iSLEEPS.

Table 3 presents the ISRUC-S3 class distribution. Relative to iSLEEPS, ISRUC-S3 contains larger proportions of N3 (22.9% versus 8.6%) and REM (12.5% versus 7.6%) and a smaller proportion of Wake (20.4% versus 30.4%). These distributional differences, together with changes in population, site, montage, sampling rate, and hardware, make the zero-shot evaluation a test under simultaneous domain shifts.

### 3.2. Data Preprocessing

**Figure 2:**
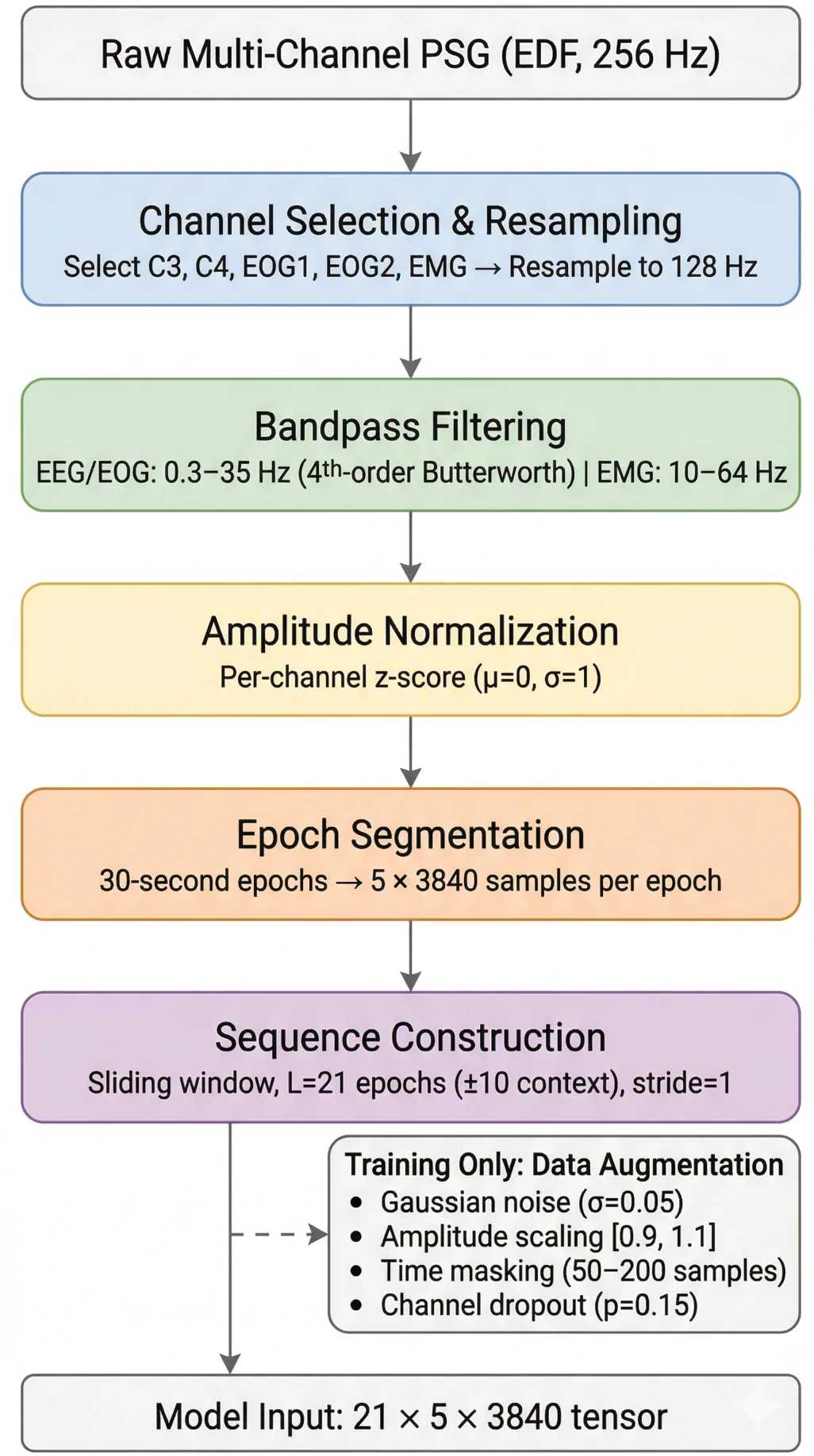
End-to-end preprocessing pipeline. Raw multi-channel PSG signals undergo channel selection, resampling to 128 Hz, bandpass filtering (0.3–35 Hz for EEG/EOG; 10–64 Hz for EMG), per-channel *z*-score normalization, 30 s epoch segmentation, and sliding-window sequence construction (L=21). Solid arrows denote the standard preprocessing path, whereas the dashed arrow denotes the training-only augmentation branch. Data augmentation (Gaussian noise, amplitude scaling, time masking, channel dropout) is applied during training only.

Figure 2 summarizes the common signal-level preprocessing applied to both datasets. Dataset-specific channel mapping, resampling, and sequence boundary handling are described below and in Section 3.5.

The raw PSG signals undergo the following common preprocessing steps, with dataset-specific channel mapping, resampling, and boundary handling described below:

1. Channel Selection and Resampling: Five signals (C3, C4, EOG1, EOG2, and EMG) are selected and resampled to 128 Hz. iSLEEPS is downsampled from 256 Hz; ISRUC is resampled from 200 Hz by polyphase rational resampling with a 16:25 ratio. The resulting Nyquist frequency is 64 Hz.
2. Bandpass Filtering: For EEG and EOG channels, the 4th-order Butterworth filter is used to bandpass filter the channels between 0.3 Hz and 35 Hz, with zero-phase forward–backward filtering (filtfilt) applied to avoid phase distortion. This frequency range keeps sleep-related oscillations while removing DC drift and high-frequency noise. The sleep-related oscillations include the delta activity (0.5–4 Hz) predominant in N3, the theta waves (4–8 Hz) characteristic of N1, the alpha rhythm (8–13 Hz) associated with relaxed wakefulness, sigma/spindle activity (12–15 Hz) marking N2, and the beta activity (15–30 Hz) associated with arousal. For the EMG channel, bandpass filtering is applied between 10 Hz and the Nyquist frequency to ensure that the tonic and phasic muscle activity patterns are captured to help distinguish between REM atonia and Wake muscle tone.
3. Amplitude Normalization: Each channel is normalized independently to a zero mean and a unit variance (*z*-score normalization) across the recording. This normalization per recording and per channel is meant to reduce the inter-subject variability in amplitude related to differences in electrode impedances, skin conductance, and recording machines while preserving the relative intra-recording dynamics and the relationships across channels.
4. Epoch Segmentation: The continuous signal is split into 30 s epochs, in accordance with the AASM annotations, leading to feature vectors of dimension 5×3840 (5 channels × 128 Hz × 30 s) per epoch. Epochs with annotations showing artifacts or movement time are removed.
5. Sequence Construction: A stride-1 sliding window creates sequences of L=21 epochs, giving the central target epoch up to 10 preceding and 10 following epochs of context. At iSLEEPS recording boundaries, unavailable positions are zero-padded and excluded from self-attention through a binary padding mask. The 21-epoch window (10.5 min) is fixed before the main cross-validation to balance local transition context and computation. The L=11 and L=31 pilot results in Section 4.5 are reported as sensitivity estimates rather than evidence of superiority.

During training only, the following stochastic augmentations are applied independently to each sampled sequence:

- Gaussian Noise Injection: With probability 0.50, additive Gaussian noise with σ=0.05 is applied to valid sequence positions.
- Random Amplitude Scaling: With probability 0.50, the sequence is multiplied by a factor sampled uniformly from [0.9,1.1].
- Random Time Masking: With probability 0.30, a randomly located segment of 50–200 samples (0.39–1.56 s at 128 Hz) is set to zero in each valid epoch.
- Random Channel Dropout: With probability 0.15, one randomly selected channel is set to zero across the sequence, encouraging robustness to a missing signal.

These augmentation methods are designed to simulate the electrode artifacts and signal variability inherent to clinical PSG recordings [22]. Data augmentation is used only in the training phase of the model, whereas validation and testing are carried out without any data augmentation.

### 3.3. Proposed Architecture: SleepStageNet

**Figure 3:**
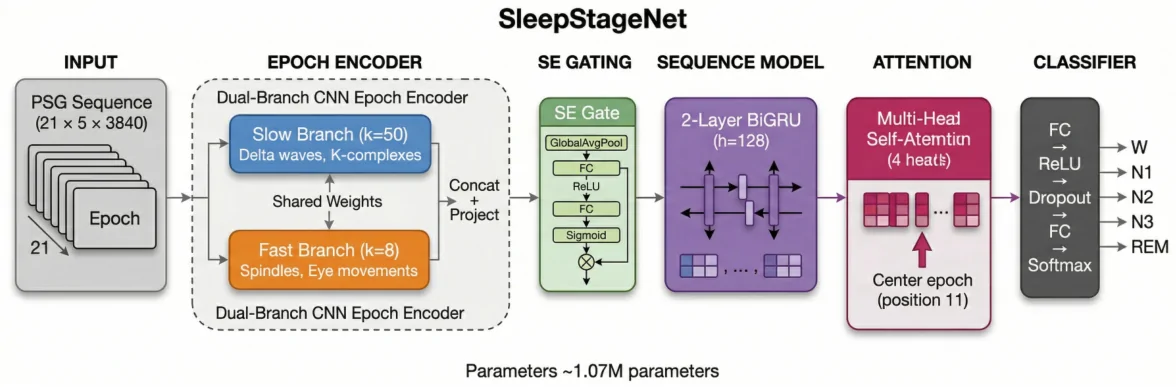
SleepStageNet processes 21 consecutive 30 s epochs (5 channels × 3840 samples) with a shared dual-branch encoder. Arrows indicate information flow, and the different colors distinguish the input, epoch-encoder, feature-gating, sequence-modeling, attention, and classifier modules. The k=50 and k=8 first-layer kernels provide different temporal receptive fields. Gated concatenated features enter a two-layer BiGRU and four-head self-attention module; the center representation is classified as W, N1, N2, N3, or REM. The executed model has 1,075,269 trainable parameters.

SleepStageNet analyzes L=21 consecutive PSG epochs and predicts the stage of the central epoch. Figure 3 shows four components adapted from prior architectures: (1) a dual-branch CNN epoch encoder, (2) feature gating, (3) a bidirectional GRU, and (4) multi-head self-attention followed by a classification layer. The compact integration, rather than an individual operator, is evaluated in this study. Algorithm 1 gives the forward pass, and Table 4 reports the executed layer configuration.

Each 30 s PSG epoch $x_{t}\in R^{C\times S}$ (where C=5 channels and S=3840 samples) is independently processed by a dual-branch CNN to capture multi-scale temporal features. The motivation behind the dual-branch design comes from the variety of sleep characteristics of clinical importance that occur over different temporal scales: slow-wave activities and K-complexes are patterns that last for hundreds of milliseconds, whereas sleep spindles as well as rapid eye movement events are transient events lasting 0.5–2 s.

The slow branch of the network is designed to capture low-frequency patterns such as K-complexes and slow-wave activities:

(1) $$h_{t}^{\mathrm{slow}}=f_{\mathrm{slow}}\left(x_{t}\right)=\mathrm{Pool}\left(\sigma (\mathrm{BN}\left({\mathrm{Conv}1d}_{k=50,s=6}\left(x_{t}\right)\right))\right)$$

where ${\mathrm{Conv}1d}_{k,s}$ denotes a 1-D convolution with kernel size *k* and stride *s*, BN(·) is batch normalization [23], σ(·) is GELU activation, and Pool(·) denotes pooling. The slow branch uses convolutional kernels of 50 and 8 samples. At 128 Hz, the first kernel spans approximately 390 ms and provides a broader temporal receptive field.

The fast branch utilizes miniature filters to capture high-frequency patterns like sleep spindles, vertex sharp waves, and rapid eye movements:

(2) $$h_{t}^{\mathrm{fast}}=f_{\mathrm{fast}}\left(x_{t}\right)=\mathrm{Pool}\left(\sigma (\mathrm{BN}\left({\mathrm{Conv}1d}_{k=8,s=2}\left(x_{t}\right)\right))\right)$$

The small kernel size of 8 samples at 128 Hz corresponds to about 62.5 ms, which is good for detecting sharp transients and high-frequency modulations in the sigma (12–15 Hz) and beta (15–30 Hz) bands. Both branches produce 128-dimensional feature maps, which are then flattened and concatenated:

(3) $$h_{t}^{\mathrm{cat}}=\left[\mathrm{Flatten}\left(h_{t}^{\mathrm{slow}}\right)\;∥\;\mathrm{Flatten}\left(h_{t}^{\mathrm{fast}}\right)\right]$$

The feature-gating mechanism, adapted from SE-Net [24], first reweights the 256 concatenated features:

(4) $$z=\sigma_{2}\left(W_{2}\cdot \sigma_{1}\left(W_{1}\cdot h_{t}^{\mathrm{cat}}\right)\right)$$

where $W_{1}\in R^{(d/r)\times d}$ and $W_{2}\in R^{d\times (d/r)}$ are learned weight matrices with reduction ratio r=4, $\sigma_{1}\left(\cdot \right)$ is GELU, and $\sigma_{2}\left(\cdot \right)$ is sigmoid. The gated output is

(5) $${\tilde{h}}_{t}=z⊙h_{t}^{\mathrm{cat}}$$

where ⊙ denotes element-wise multiplication. A linear projection then maps the gated vector to d=256 dimensions:

(6) $$e_{t}=W_{\mathrm{proj}}{\tilde{h}}_{t}+b_{\mathrm{proj}},\;e_{t}\in R^{d}.$$

Sleep staging has fundamental temporal dependencies due to the sequencing of physiological events (e.g., direct Wake→REM transitions are rarely seen outside narcolepsy, and N3 epochs tend to cluster in the first half of the night). Inter-epoch temporal dependencies are captured by a 2-layer bidirectional GRU [25]. Given the projected sequence $\left\{e_{1},e_{2},\ldots ,e_{L}\right\}$, the BiGRU computes

(7) $$\begin{array}{cc} {\vec{h}}_{t} & ={\mathrm{GRU}}_{\mathrm{fwd}}\left(e_{t},{\vec{h}}_{t-1}\right) \end{array}$$

(8) $$\begin{array}{cc} {\overset{\leftarrow }{h}}_{t} & ={\mathrm{GRU}}_{\mathrm{bwd}}\left(e_{t},{\overset{\leftarrow }{h}}_{t+1}\right) \end{array}$$

(9) $$\begin{array}{cc} g_{t} & =\left[{\vec{h}}_{t}\;∥\;{\overset{\leftarrow }{h}}_{t}\right] \end{array}$$

where ${\vec{h}}_{t},{\overset{\leftarrow }{h}}_{t}\in R^{128}$ are the forward and backward hidden states and $g_{t}\in R^{256}$. Two stacked layers use dropout 0.3 between layers. The bidirectional formulation allows the central prediction to use both the preceding and following context within the 21-epoch window.

To improve temporal representations and allow the model to learn dynamic attention patterns over the epoch sequence, multi-head self-attention [26] is applied. Given the GRU output sequence $G=\left[g_{1},\ldots ,g_{L}\right]\in R^{L\times d}$,

(10) $$\mathrm{Attention}\left(Q,K,V\right)=\mathrm{softmax}\left(\frac{QK^{⊤}}{\sqrt{d_{k}}}\right)V$$

where queries $Q=GW^{Q}$, keys $K=GW^{K}$, values $V=GW^{V}$ are linear projections, and $d_{k}=d/n_{\mathrm{heads}}$ is the per-head dimension. The model uses $n_{\mathrm{heads}}=4$ attention heads with $d_{k}=64$. To stabilize training and preserve the temporal representations from the BiGRU, a residual connection with layer normalization is applied after the attention layer.

The self-attention output for the center epoch (position ⌊L/2⌋) is extracted and passed to the classification head.

The classification head consists of dropout followed by a fully connected layer:

(11) $$\hat{y}=\mathrm{softmax}\left(W_{c}\;\mathrm{Dropout}\left(a_{\left\lfloor L/2\right\rfloor }\right)+b_{c}\right)$$

where $a_{\left\lfloor L/2\right\rfloor }\in R^{256}$ is the attention output for the center epoch, $W_{c}\in R^{5\times 256}$, and dropout 0.5 is applied before the output layer.
**Algorithm 1** SleepStageNet Forward Pass**Require:** Sequence of L=21 PSG epochs $X=[x_{1},\ldots ,x_{L}]$, each $x_{t}\in R^{5\times 3840}$
**Ensure:** Predicted class probabilities $\hat{y}\in R^{5}$
  1:**for** t=1 to *L* **do**  2:      $h_{t}^{\mathrm{slow}}\leftarrow$SlowBranch$\left(x_{t}\right)$                                                                   // Large kernel CNN  3:      $h_{t}^{\mathrm{fast}}\leftarrow$FastBranch$\left(x_{t}\right)$                                                                      // Small kernel CNN  4:      ${\tilde{h}}_{t}\leftarrow$FeatureGate$\left(\mathrm{Concat}\left(h_{t}^{\mathrm{slow}},h_{t}^{\mathrm{fast}}\right)\right)$                                       // Feature recalibration  5:      $e_{t}\leftarrow$Project$\left({\tilde{h}}_{t}\right)$                                                                                                         // $R^{256}$  6:**end for**  7:G←BiGRU$\left(\left[e_{1},\ldots ,e_{L}\right]\right)$                                                                            // Temporal context  8:A←MultiHeadAttn(G,G,G)                                                                       // Self-attention  9:$\hat{y}\leftarrow$Classifier$\left(A_{\left\lfloor L/2\right\rfloor }\right)$                                                                                      // Center epoch10:**return** $\hat{y}$


### 3.4. Training Strategy

A 10-fold stratified group cross-validation scheme is used with subjects as the grouping variable. All epochs from a subject occur in either training or validation within a fold, never both. Across the 95 analyzed subjects, folds contain 84–86 training subjects and 9–11 validation subjects. Stratification approximately preserves stage distributions while grouping prevents intra-subject leakage.

Class-weighted cross-entropy with label smoothing [27] is used:

(12) $$L=-\sum_{c=1}^{5}w_{c}\;{\tilde{y}}_{c}\log\left({\hat{y}}_{c}\right)$$

where ${\tilde{y}}_{c}=\left(1-\epsilon \right)y_{c}+\epsilon /5$, ϵ=0.1, and $w_{c}$ is the inverse-frequency class weight. The executed weights for W, N1, N2, N3, and REM are 0.418, 1.140, 0.301, 1.479, and 1.662, respectively. The same weights and smoothing factor are used in every fold and matched comparison.

AdamW [28] is used with maximum learning rate $10^{-3}$ and weight decay $10^{-2}$. A OneCycleLR schedule [29] increases the learning rate during the first 10% of batch updates and applies cosine annealing thereafter. Training is capped at 60 epochs with batch size 128, gradient norm clipping at 1.0, mixed precision, and early stopping after 20 epochs without improvement in validation κ. The checkpoint with the highest validation κ is retained.

The hyperparameters are fixed across folds and variants rather than tuned separately on validation folds. The 256-dimensional epoch representation, 128 hidden units per GRU direction, and four attention heads are chosen to maintain a roughly one-million-parameter budget while keeping the attention dimension divisible across heads. Dropout (0.3 in the encoder and recurrent stack; 0.5 before classification), weight decay, label smoothing, and augmentation are used together because the analyzed cohort is small and class-imbalanced. The 21-epoch window provides 10 epochs of context on each side of the target while retaining batch size 128 on the available GPU. Pilot sequence length estimates are reported in Section 4.5 but are not used for fold-specific selection.

### 3.5. External Validation Protocol

**Figure 4:**
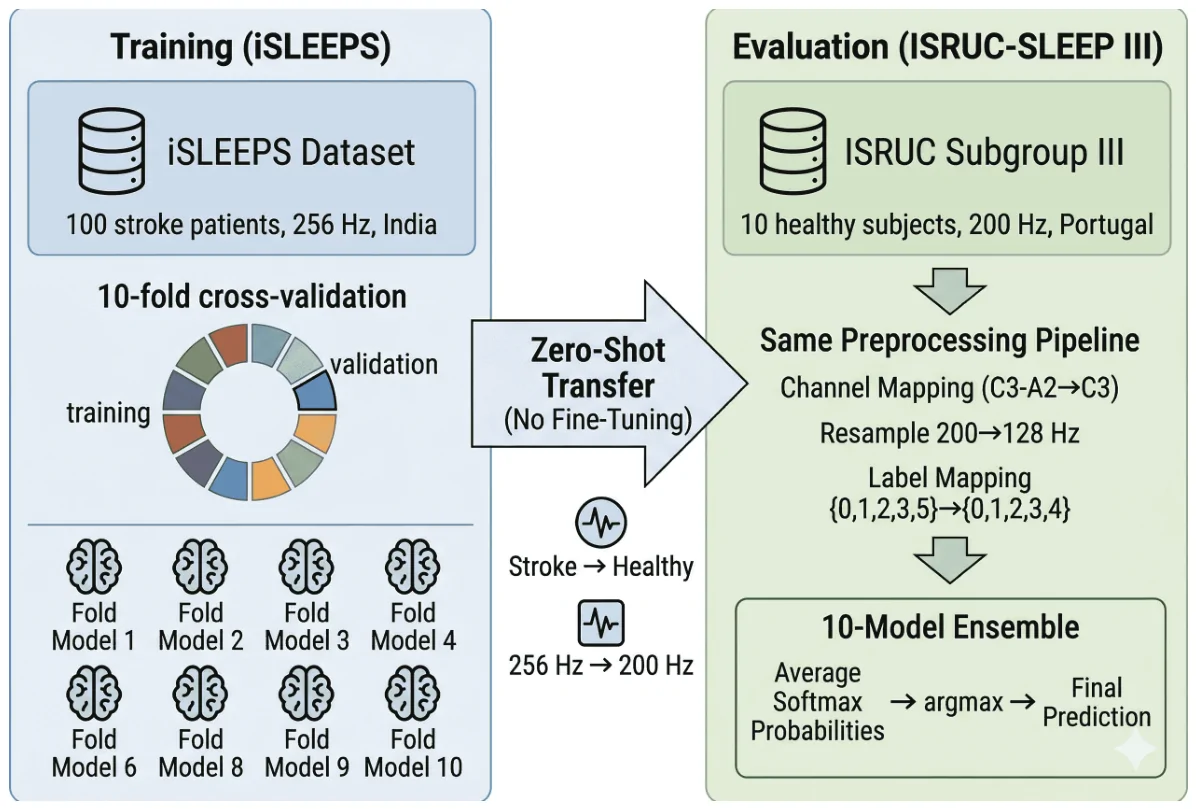
Cross-dataset protocol. The blue and red panels distinguish the iSLEEPS training domain and ISRUC-SLEEP external evaluation domain, respectively; the central arrow denotes zero-shot transfer without fine-tuning. Ten fold models trained on the 95 analyzed iSLEEPS recordings (acute stroke cohort; 256 Hz; SOMNOmedics) are evaluated without fine-tuning on ISRUC-SLEEP Subgroup III (10 healthy subjects; 200 Hz; Hospital of Coimbra). ISRUC channels and labels are mapped to the study representation, signals are resampled to 128 Hz, and softmax probabilities are averaged across models.

Figure 4 illustrates the cross-dataset evaluation pipeline.

To examine cross-dataset generalizability, the 10 best model checkpoints (one per fold) trained on the iSLEEPS dataset are assessed on the ISRUC-SLEEP Subgroup III data without domain adaptation or fine-tuning. The ISRUC data undergoes the same preprocessing pipeline described in Section 3.2, with the following adaptations:

- Channel Mapping: ISRUC channels C3-A2, C4-A1, LOC-A2, ROC-A1, and X1 are mapped to C3, C4, EOG1, EOG2, and EMG, respectively. Differences in the reference montage can alter signal amplitude and morphology and are treated as one component of the domain shift.
- Resampling: To match the iSLEEPS preprocessing pipeline, ISRUC signals are resampled from 200 Hz to 128 Hz using polyphase rational resampling (factor 16:25).
- Label Mapping: ISRUC labels {0,1,2,3,5} are transformed to the standard 5-class indices {0,1,2,3,4}.
- Sequence Boundaries: For the first and last 10 epochs of each ISRUC recording, the nearest available epoch is repeated to complete the 21-epoch input window.

Each of the 10 fold models generates independent predictions on the entire ISRUC evaluation set. An ensemble prediction is computed by averaging the softmax probability vectors across all 10 models before taking the argmax, following the standard model averaging strategy. This ensemble methodology takes advantage of the diversity among models trained on different subject partitions to produce more robust predictions.

### 3.6. Evaluation Metrics

In light of the significant class imbalance (Table 2), a comprehensive set of evaluation metrics is employed:

- Overall Accuracy: The proportion of epochs that are correctly classified.
- Macro F1-Score: The unweighted average of per-class F1-scores for each class, treating all classes equally regardless of their frequency:
  (13) $${F1}_{\mathrm{macro}}=\frac{1}{K}\sum_{c=1}^{K}\frac{2\cdot P_{c}\cdot R_{c}}{P_{c}+R_{c}}$$
  where $P_{c}$ and $R_{c}$ represent the precision and recall for class *c* and K=5.
- Cohen’s Kappa (κ) [30]: A chance-corrected agreement metric:
  (14) $$\kappa =\frac{p_{o}-p_{e}}{1-p_{e}}$$
  where $p_{o}$ is the observed agreement and $p_{e}$ is the expected agreement by chance. Values of κ in the range 0.61–0.80 indicate “substantial agreement” per Landis and Koch’s scale.
- Per-Class Precision, Recall, and F1-Score: Used to evaluate performance on each sleep stage independently, which is essential given the clinical importance of correctly identifying all stages.

### 3.7. Statistical Analysis

For the primary 10-fold evaluation, means, standard deviations, and two-sided 95% confidence intervals are reported across subject-independent validation folds; confidence intervals use the Student’s *t*-distribution with nine degrees of freedom. Two comparisons are specified in response to the architectural questions: the full model versus (i) the five-channel single-branch encoder and (ii) the full architecture with C4 input only. Both variants use the same subject folds and 60-epoch training protocol as the full model. For C4-only training, Gaussian noise, amplitude scaling, and time masking are retained, while channel dropout augmentation is disabled because dropping the sole channel would erase the complete input. Fold-level κ values are compared with two-sided Wilcoxon signed-rank tests, with Holm adjustment across the two tests. The mean paired difference and rank–biserial correlation are reported as effect size summaries. Because cross-validation training sets overlap, these tests are interpreted together with fold-level confidence intervals rather than as independent-sample evidence. Published results from Bose et al. and ISRUC-trained models use different splits or training domains and are therefore compared descriptively, without significance claims.

## 4. Results and Analysis

Table 5 summarizes the subject-independent 10-fold results for the 95 analyzed iSLEEPS recordings. Mean accuracy is 73.91% ± 2.24% (95% CI: 72.31–75.51%), the macro F1-score is 67.29% ± 1.96% (95% CI: 65.89–68.70%), and Cohen’s κ is 0.634±0.029 (95% CI: 0.613–0.654). Variability is reported as the sample standard deviation across folds. The point estimate lies in the “substantial agreement” interval of the Landis–Koch descriptive scale; this scale does not by itself establish clinical utility.

**Figure 5:**
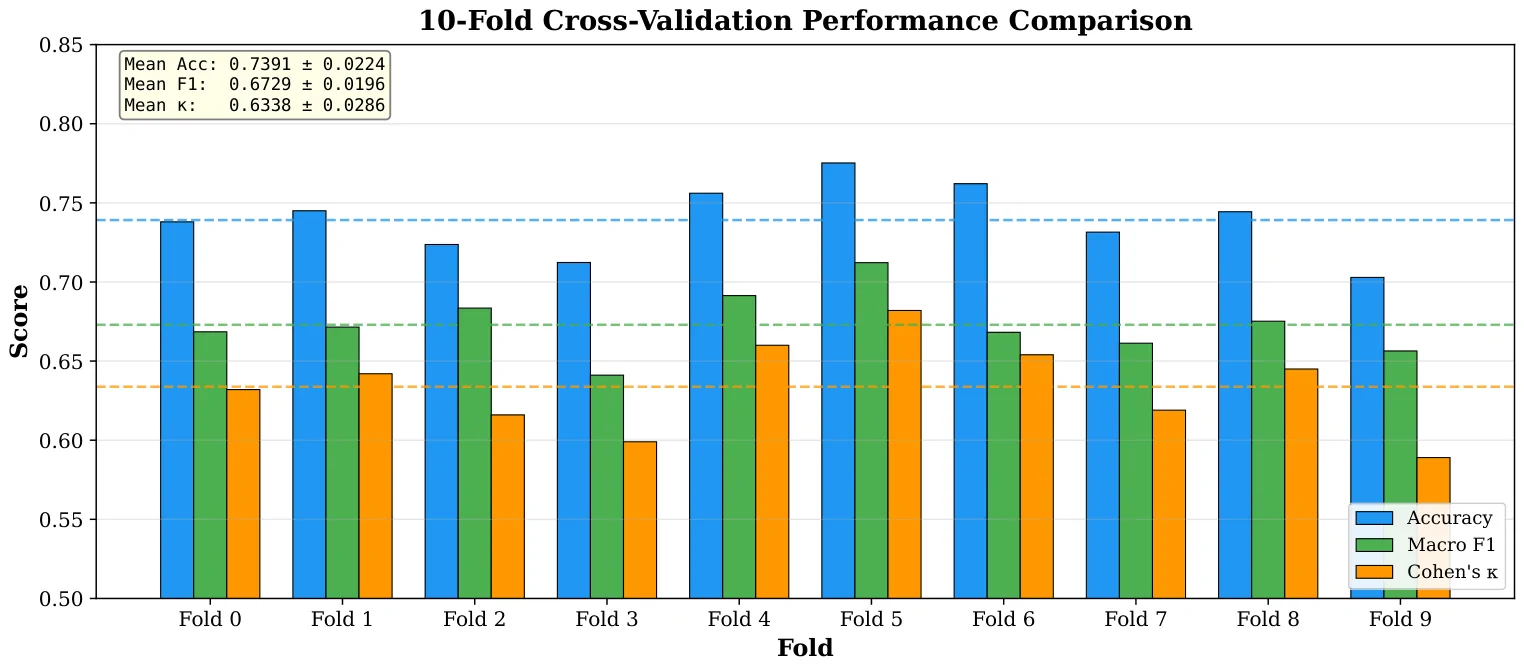
Accuracy, macro F1-score, and Cohen’s κ across the 10 subject-independent validation folds. Dashed lines denote means; the spread reflects sensitivity to held-out subject composition and is summarized by confidence intervals in Table 5.

Figure 5 shows all fold-level metrics. Cohen’s κ ranges from 0.589 (Fold 9) to 0.682 (Fold 5), with a sample standard deviation of 0.029. This variability is retained in the confidence intervals and matched fold-level comparisons.

### 4.1. Per-Class Performance

Table 6 reports per-class results averaged across folds. Wake and N2 have the highest F1-scores (0.822 and 0.801), N3 and REM reach 0.698 and 0.649, and N1 has the lowest value (0.395). Class frequency and stage morphology can both affect these differences, but the aggregate metrics do not isolate their respective contributions.

**Figure 6:**
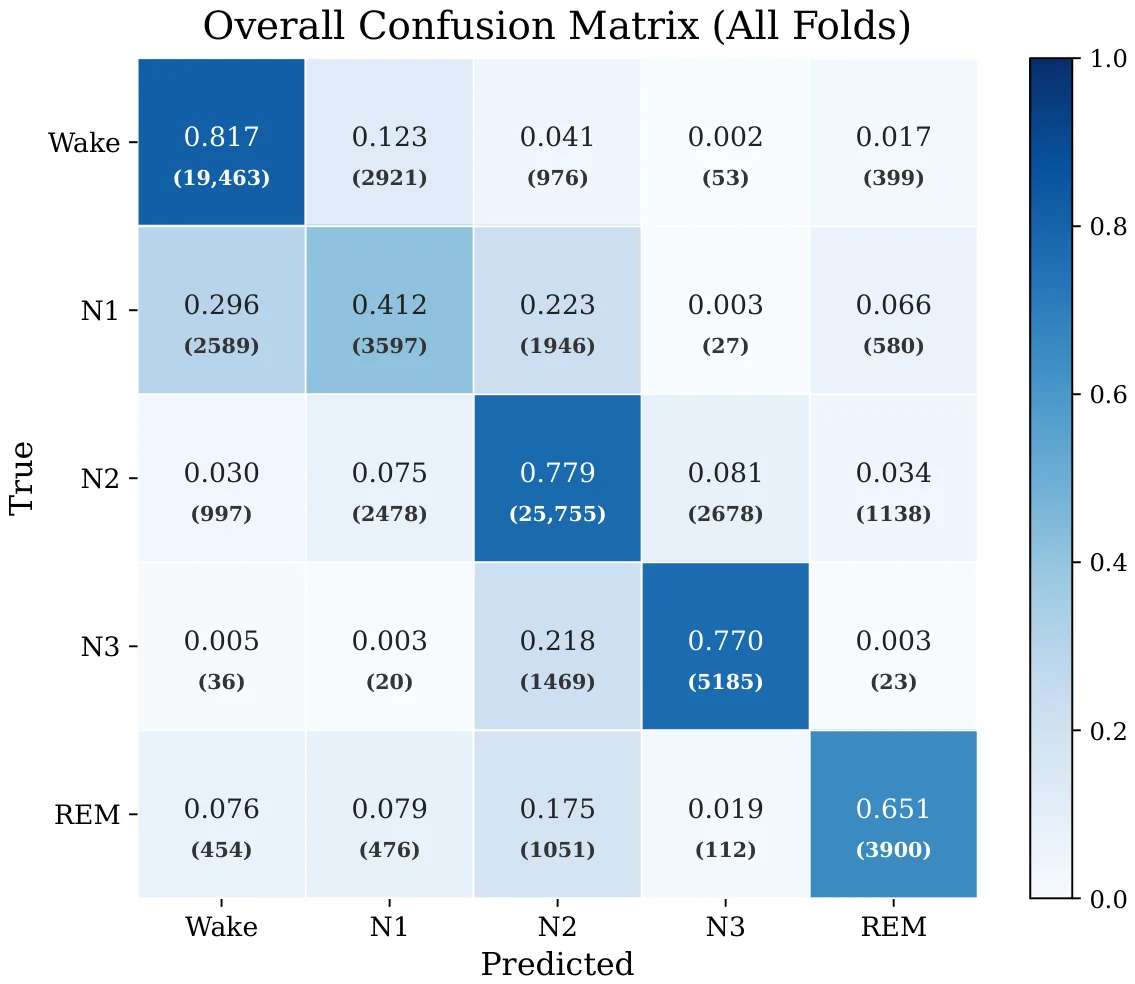
Overall confusion matrix aggregated across all 10 cross-validation folds. N1 is the most challenging class, with significant confusion with Wake and N2. The diagonal values indicate correct classifications.

Figure 6 aggregates out-of-fold predictions. N1 is predicted as Wake for 29.6% of N1 epochs and as N2 for 22.3%, consistent with ambiguity at adjacent stage boundaries. N2 and N3 also overlap: 8.1% of N2 epochs are predicted as N3, and 21.8% of N3 epochs are predicted as N2.

### 4.2. Learning Dynamics

**Figure 7:**
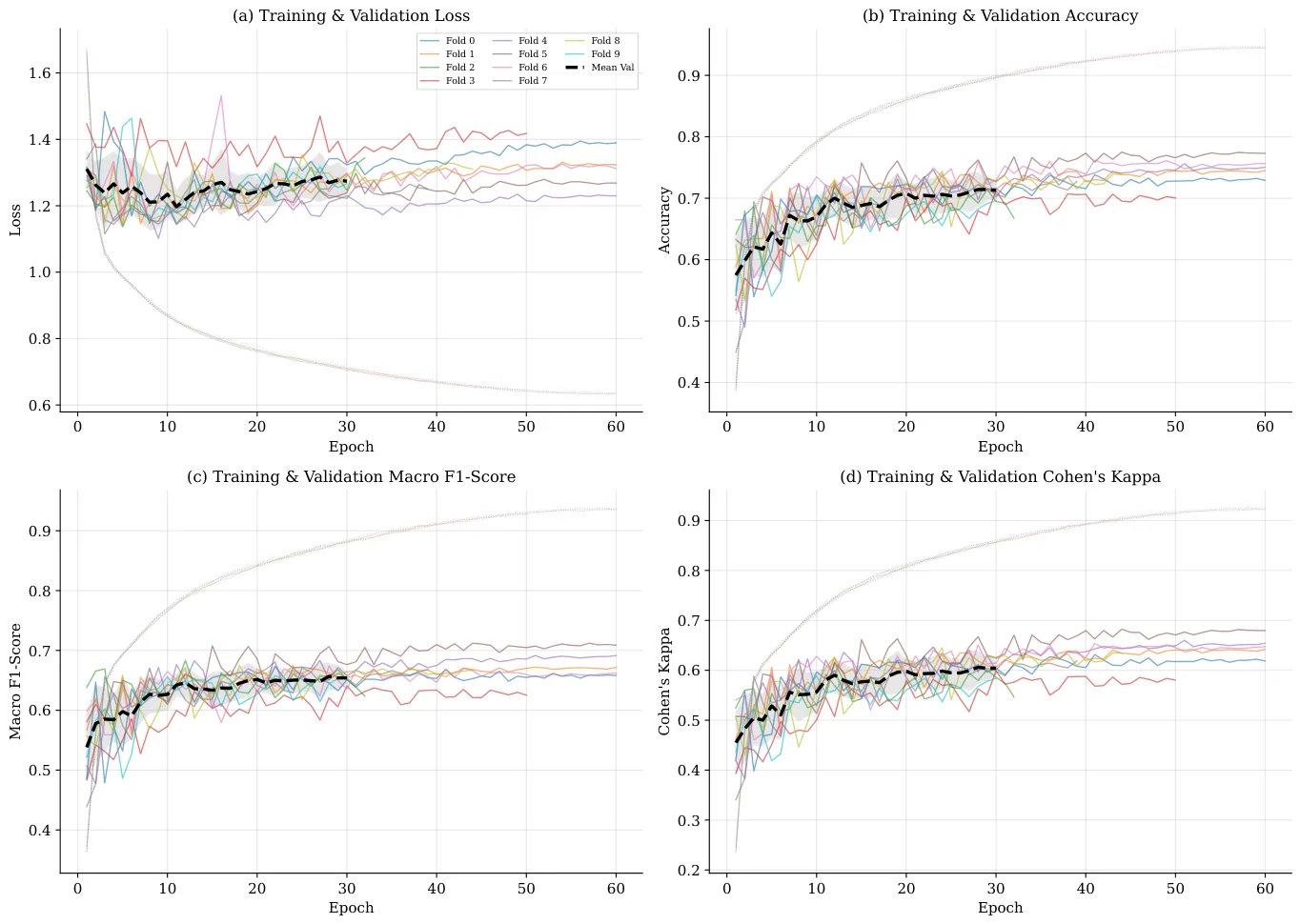
Training and validation curves across all 10 folds. (**a**) Loss, (**b**) accuracy, (**c**) macro F1-score, (**d**) Cohen’s kappa. Individual fold curves are shown in color; the mean validation curve with ±1 standard deviation is shown in black.

Figure 7 presents training and validation loss, accuracy, macro F1-score, and κ across the 10 folds. Fold-specific best epochs vary from 9 to 59, reflecting heterogeneous validation subjects. The OneCycleLR increasing phase covers the first 10% of batch updates, followed by cosine annealing.

### 4.3. Computational Profile

Table 7 reports a reproducible forward-pass benchmark. Each input is one 21-epoch context window. Timing excludes data loading and host-to-device transfer and follows warm-up iterations. CPU measurements use an Intel Xeon E5-2686 v4; GPU measurements use automatic mixed precision on an NVIDIA RTX 2080 Ti with PyTorch 2.12.0 and CUDA 13.0. The model can therefore run without a GPU, while GPU batching increases throughput. These measurements establish the computational feasibility on the tested hardware but are not a substitute for prospective workflow evaluation.

### 4.4. ROC and Precision–Recall Analysis

**Figure 8:**
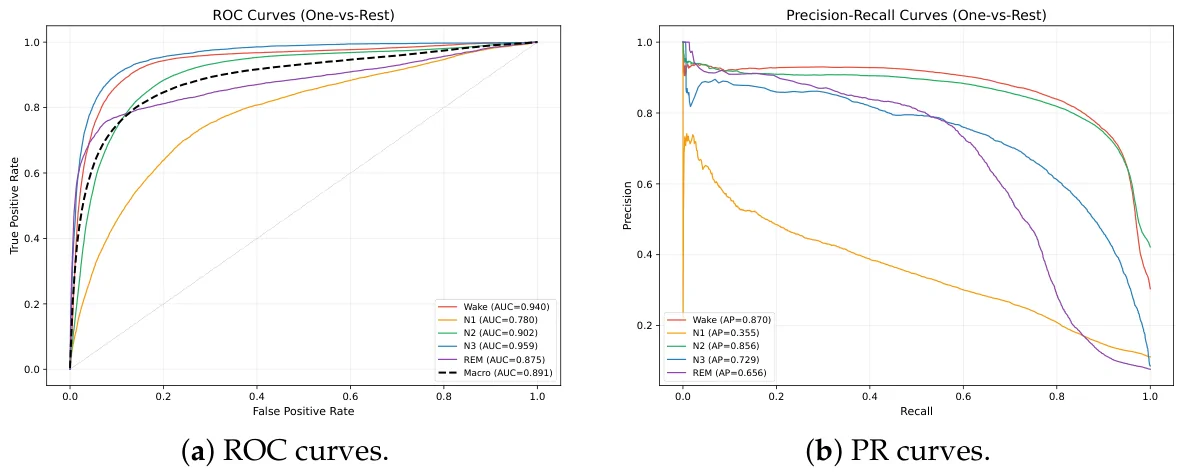
One-versus-rest (**a**) ROC and (**b**) precision–recall curves from aggregated out-of-fold predictions. The gray diagonal in panel (**a**) denotes chance-level ROC performance. PR curves retain sensitivity to class prevalence; N1 and REM show the lowest precision–recall performance.

Figure 8 presents receiver operating characteristic (ROC) and precision–recall (PR) curves computed as one versus rest. Wake and N3 have the highest ROC AUC values (0.940 and 0.959, respectively), whereas N1 has the lowest value (0.780). The PR curves show lower precision–recall performance for the minority N1 and REM classes.

### 4.5. Ablation Study

An exploratory component screen is conducted on validation Fold 0 with a maximum of 30 training epochs and an early-stopping patience of 12. Each variant uses the same fold and pilot training budget. Because this screen contains one run per variant, its point estimates are not used for significance claims. Table 8 reports $\Delta \kappa =\kappa_{\mathrm{variant}}-\kappa_{\mathrm{full}}$, so negative values denote lower performance than the pilot full model.

Within this pilot, removing the BiGRU gives the largest point estimate change (Δκ=−0.089). The single-branch model is slightly higher than the pilot full model (Δκ=+0.007), whereas removal of feature gating (−0.001), the sequence length changes (−0.003 and −0.006), and removal of augmentation (−0.004) are small relative to the between-fold variability of the main model. Accordingly, the pilot supports further testing of the recurrent component but does not establish benefits for feature gating, dual-branch encoding, augmentation, or L=21. The dual- versus single-branch question is evaluated under the complete 60-epoch, 10-fold protocol below.

**Figure 9:**
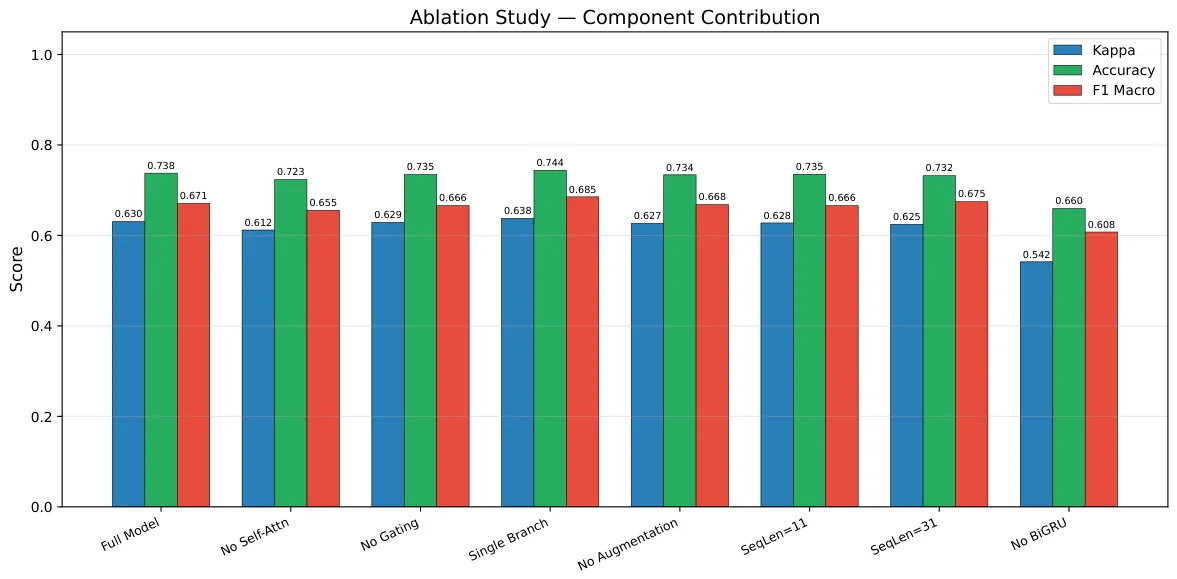
Exploratory Fold 0 component screen with a maximum of 30 epochs. Removing the BiGRU produces the largest point estimate decrease; the remaining small differences are not interpreted as statistically established because each variant is run once in this pilot.

Figure 9 presents a visual comparison of the ablation variants.

### 4.6. Full-Protocol Matched Comparisons

Table 9 reports the two reviewer-requested variants trained on all 10 subject folds with the complete protocol. The five-channel single-branch encoder produces nearly the same estimates as the full dual-branch model: κ=0.635 versus 0.634. The C4-only full architecture obtains κ=0.600.

Table 10 reports the corresponding prespecified paired statistical comparisons.

For the dual- versus single-branch comparison, the paired difference includes zero and the Holm-adjusted test is not significant (p=0.846; rank-biserial correlation $r_{\mathrm{rb}}=-0.091$). The study therefore does not establish a performance benefit from the additional CNN branch. In contrast, the five-channel full model exceeds its matched C4-only version by mean Δκ=0.0333 (95% CI: 0.0098–0.0569; Holm-adjusted p=0.0078; $r_{\mathrm{rb}}=0.964$), supporting a benefit from the additional PSG signals within this pipeline. This matched result is not a significance comparison with the results of Bose et al., whose single-channel point estimates remain slightly higher under a different protocol.

### 4.7. Explainability Analysis

Three complementary post hoc analyses are used to characterize SleepStageNet: Grad-CAM, temporal attention visualization, and channel occlusion. These analyses are performed with the best Fold 0 model and its validation data. They describe model sensitivity but do not constitute prospective clinical validation or proof of a physiological mechanism.

**Figure 10:**
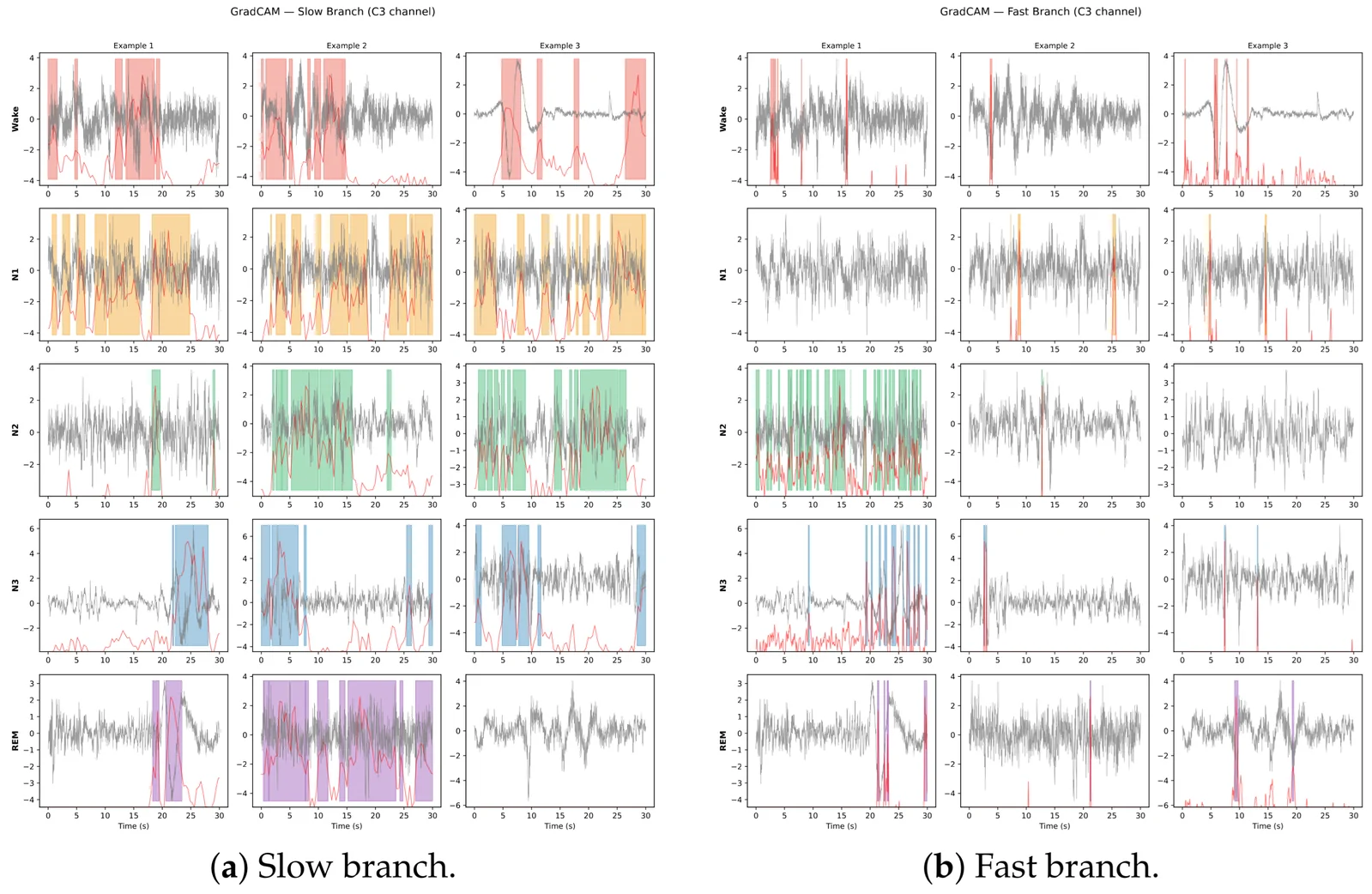
Representative Grad-CAM overlays from the Fold 0 model for the (**a**) slow and (**b**) fast encoder branches. Gray traces show the C3 signal, red curves and shading show normalized Grad-CAM saliency, and the stage-specific shaded colors identify the sleep-stage rows. Greater red intensity denotes greater local sensitivity of the selected class score on the C3 trace. Broader slow-branch and more localized fast-branch activations are physiologically plausible for complementary temporal scales, but these examples do not identify or validate specific graphoelements.

Grad-CAM [31] is applied to the last convolutional layer of each branch. Figure 10 shows representative C3 traces and saliency overlays. Broad activations appear in several slow-branch examples, whereas some fast-branch maps are more localized. Their timing is compatible with sustained and transient sleep-related activity, respectively, but the displayed epochs are not independently annotated for specific graphoelements; the maps therefore remain qualitative.

**Figure 11:**
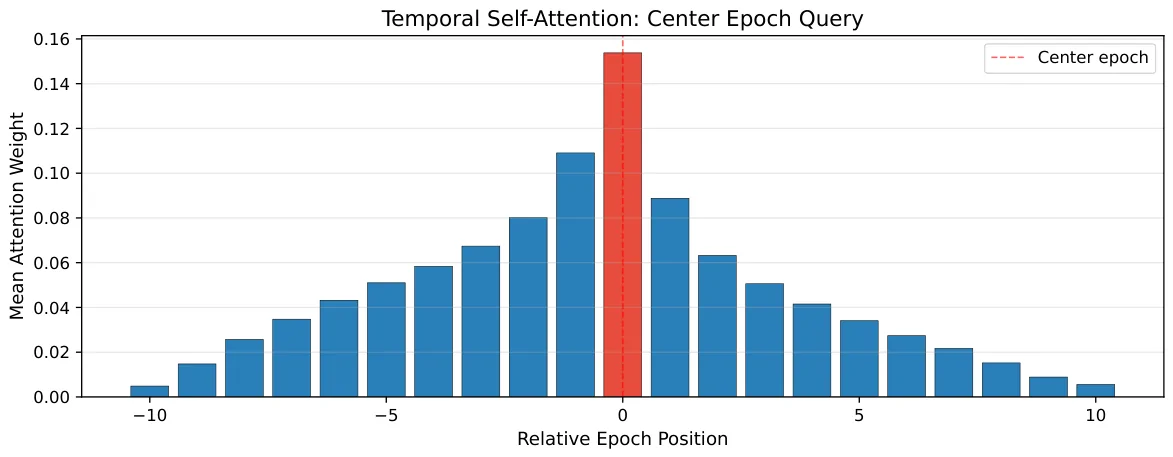
Mean Fold 0 self-attention weight from the center-epoch query to each position in the 21-epoch context (up to 50 validation batches). The red bar and dashed line identify the center target epoch, while the blue bars show the surrounding context positions. The center receives the largest weight and nearby epochs retain nonzero influence, a pattern compatible with local continuity in sleep stage transitions. Attention weight is model sensitivity, not a direct measure of biological causality.

Figure 11 summarizes the center-query attention weights across the 21-epoch sequence. The largest mean weight occurs at the center, and nearby epochs retain nonzero weights. This distribution describes the model’s allocation of attention and is not treated as a causal explanation.

**Figure 12:**
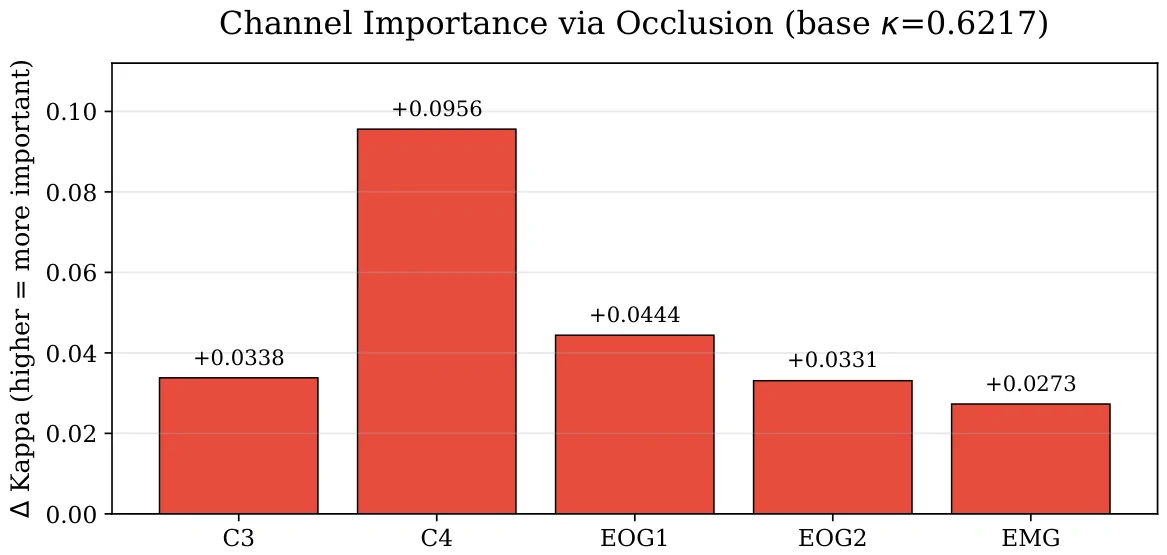
Fold 0 channel occlusion analysis (baseline κ=0.6217). Each bar is the decrease in κ after one channel is zeroed while the others are retained. C4 produces the largest decrease in this model and subset; the EEG/EOG pattern is consistent with their established role in stage scoring, but occlusion effects can include channel redundancy and should not be interpreted causally.

In the Fold 0 channel occlusion analysis, each signal is zeroed separately and the change in κ is measured. Table 11 reports the numerical values, and Figure 12 visualizes them. C4 produces the largest decrease (0.096), followed by EOG1 (0.044), C3 (0.034), EOG2 (0.033), and EMG (0.027). This ranking is compatible with the established roles of central EEG and EOG in staging, but it may also reflect redundancy among correlated channels.

### 4.8. Feature Space Visualization

**Figure 13:**
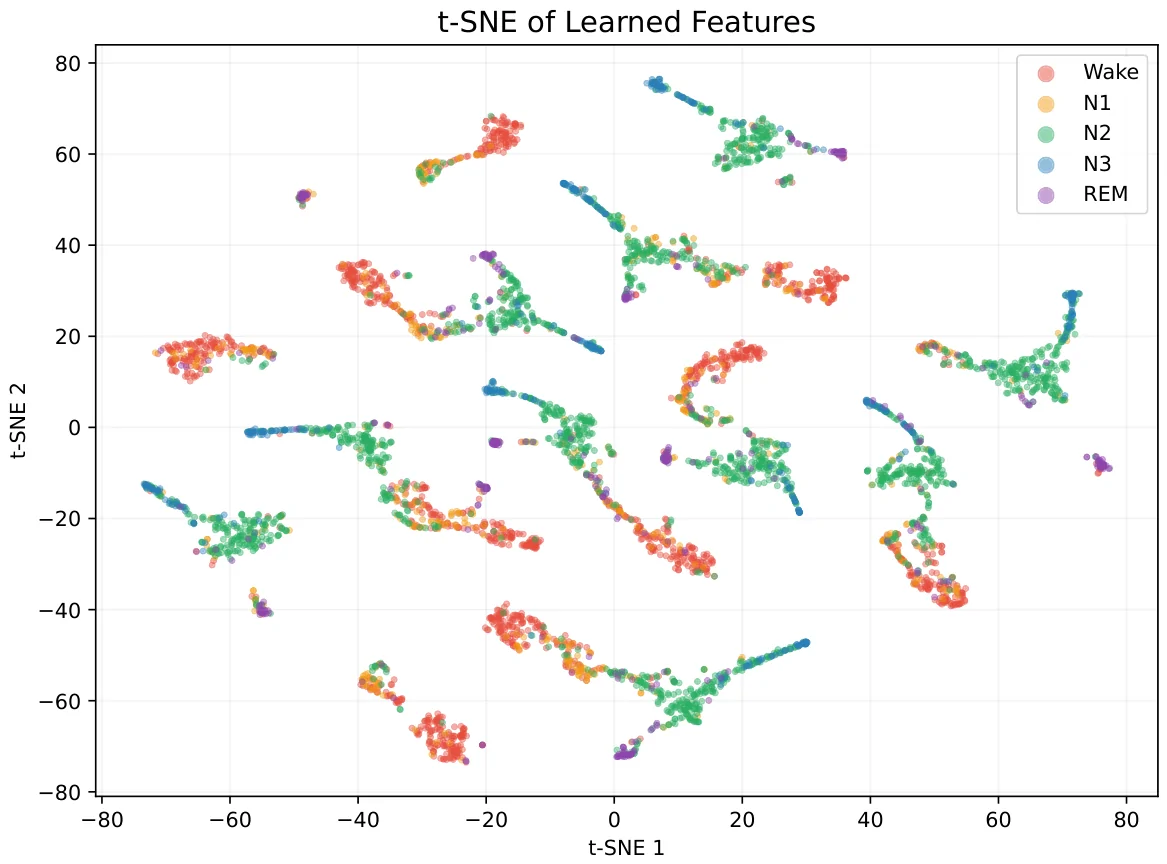
Two-dimensional t-SNE projection of penultimate-layer features. Wake, N2, and N3 are relatively separated, whereas N1 overlaps with Wake and N2, paralleling the confusion matrix and the transitional physiology of N1. Because t-SNE is a nonlinear qualitative projection, distances and cluster sizes are not quantitative evidence of class separability.

Figure 13 projects penultimate-layer features with t-SNE [32]. Wake, N2, and N3 are relatively separated in this projection, while N1 overlaps with Wake and N2 and REM overlaps partly with N1. This qualitative pattern parallels the confusion matrix; t-SNE geometry is not quantitative evidence of physiological separation.

### 4.9. External Validation on ISRUC-SLEEP Subgroup III

To evaluate the cross-dataset generalizability of SleepStageNet, the 10 models trained on the iSLEEPS dataset are assessed on the ISRUC-SLEEP Subgroup III dataset without fine-tuning. This constitutes a highly challenging test of transferability, as the training and evaluation datasets vary across multiple dimensions: population demographics (stroke patients vs. healthy subjects), recording equipment (SOMNOmedics vs. Hospital of Coimbra system), native sampling rates (256 Hz vs. 200 Hz), electrode montage conventions, and geographic/ethnic backgrounds (Indian vs. Portuguese).

Table 12 presents each iSLEEPS fold model’s performance on ISRUC. Individual κ values range from 0.546 (Fold 9) to 0.639 (Fold 4), with a mean of 0.580±0.033. The variation indicates sensitivity to the iSLEEPS training partition; it does not identify which subject characteristics caused the difference.

The 10-model ensemble, obtained by averaging softmax probabilities, reaches 70.14% accuracy, a 67.65% macro F1-score, and κ=0.614. This is 0.034 above the mean individual-model κ of 0.580, although Fold 4 (κ=0.639) and Fold 6 (κ=0.635) have higher individual point estimates. The ensemble is therefore reported as a stable aggregate rather than as uniformly superior to every constituent model.

Table 13 provides an in-domain reference for interpreting the zero-shot result. 3DSleepNet [33] and MixSleepNet [34] were trained and evaluated on ISRUC-S3 with subject-independent 10-fold cross-validation, whereas SleepStageNet received no ISRUC training or fine-tuning. Their higher κ values (0.783 and 0.782) quantify the expected advantage of in-domain training. Because the input channels, feature construction, and training data differ, these values are context rather than matched competitors.

Table 14 presents the per-class ensemble performance on ISRUC. REM and N3 have the highest F1-scores (0.794 and 0.785), while N1 has the lowest (0.374). ISRUC contains larger proportions of N3 and REM than iSLEEPS (22.9% and 12.5% versus 8.6% and 7.6%), but differences in population, montage, hardware, and scoring prevent attribution of the per-class changes to class distribution or health status alone.

Table 15 reports per-subject ensemble performance. Subject-level κ ranges from 0.485 to 0.695, with a standard deviation of 0.059. This spread shows material between-subject variation and does not support uniform performance across individuals.

**Figure 14:**
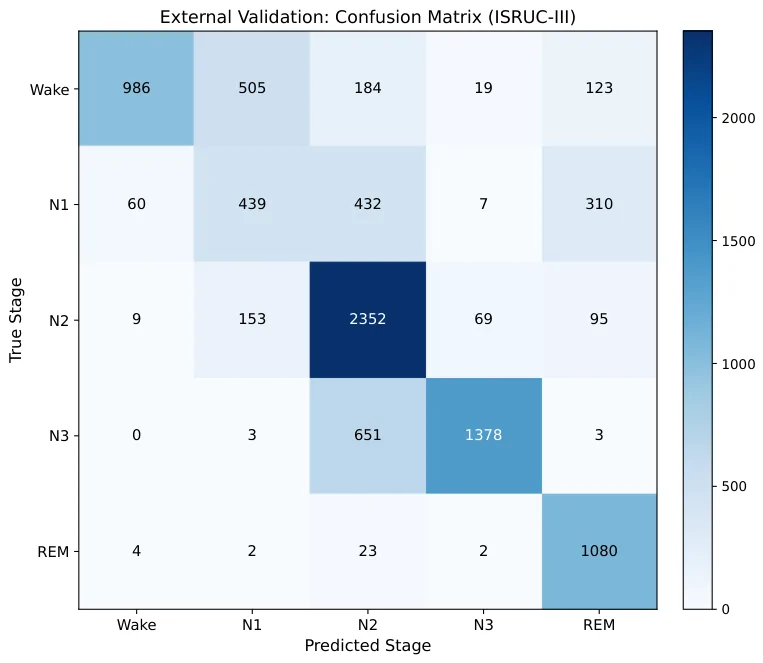
Row-normalized confusion matrix for zero-shot evaluation of the 10-model ensemble on ISRUC-SLEEP Subgroup III. REM has the highest recall; Wake and N1 show the greatest overlap with adjacent stages. Values describe this cohort and should not be interpreted as a biological mechanism.

Figure 14 shows the ISRUC ensemble confusion matrix. Wake recall is 54.3%, with 27.8% and 10.1% of Wake epochs predicted as N1 and N2. REM recall is 97.2%. N3 precision is 93.4% and recall is 67.7%, with 32.0% of N3 epochs predicted as N2. These descriptive patterns are compatible with adjacent-stage ambiguity, but the evaluation does not isolate their physiological or dataset-specific causes.

**Figure 15:**
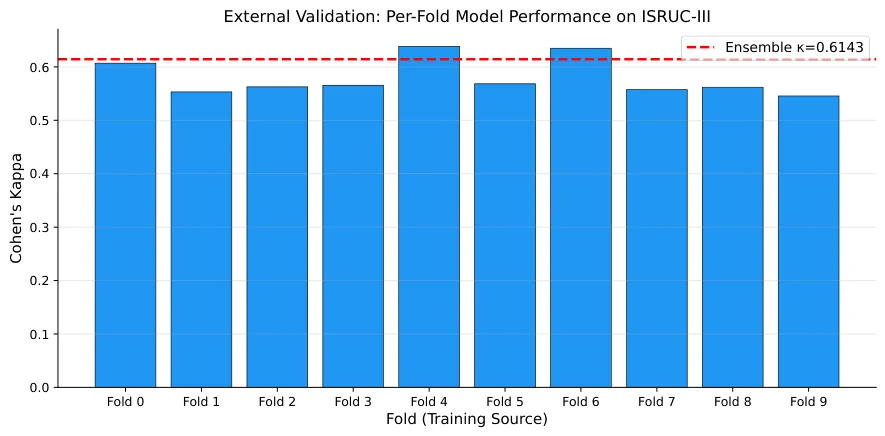
Cohen’s κ for the 10 iSLEEPS fold models evaluated zero-shot on ISRUC-SLEEP Subgroup III. The dashed line is the probability-averaged ensemble (κ=0.614); Fold 4 and Fold 6 have higher individual point estimates.

Figure 15 compares the per-fold model kappa values on ISRUC, with the ensemble kappa shown as a dashed reference line.

## 5. Discussion

In light of the unique challenges posed by this clinical dataset, the overall performance of SleepStageNet (κ=0.634, accuracy = 73.91%) on the iSLEEPS dataset needs to be contextualized. The sleep architecture of all subjects, who are acute stroke patients, is known to be disrupted by the neurological event itself along with concurrent medications (sedatives, anticoagulants, antihypertensives) and the hospital environment [11]. These factors produce atypical sleep patterns including fragmented sleep, prolonged wake-after-sleep-onset periods, reduced slow-wave sleep, and diminished REM density, all of which pose challenges to automated staging. The imbalance ratio of 5.5:1 between the most prevalent class (N2, 42.2%) and the least prevalent (REM, 7.6%) is more pronounced than in standard benchmarks such as Sleep-EDF-20, where the imbalance ratio is approximately 3.5:1. In addition, the wide age range (22–78 years), various stroke subtypes, and varying degrees of comorbid obstructive sleep apnea (AHI: 0–81.6) introduce substantial heterogeneity that complicates model generalization across subjects.

The subject-independent 10-fold protocol prevents epochs from one subject from appearing in both training and validation within a fold, avoiding the leakage associated with epoch-level splitting. Inter-scorer agreement for AASM scoring is commonly reported around κ=0.72–0.82 [4]; the model mean of 0.634 (95% CI: 0.613–0.654) remains below that reference range. A single best fold is not used to claim expert-level performance.

The N1 F1-score is 0.395. N1 is a transitional stage defined by attenuation of posterior alpha activity and the onset of low-amplitude mixed-frequency activity, so its boundaries with Wake and N2 are often ambiguous. In iSLEEPS, 29.6% of N1 epochs are predicted as Wake and 22.3% as N2, consistent with the low human agreement commonly reported for N1 [4]. ISRUC also shows low N1 performance (F1 = 0.374), indicating that this difficulty is observable in both evaluated cohorts; however, two datasets are insufficient to establish that it is dataset-independent.

The exploratory Fold 0 screen gives its largest point estimate decrease when the BiGRU is removed (Δκ=−0.089; variant minus full pilot). This finding is compatible with a contribution from inter-epoch context, but one pilot fold cannot establish the magnitude or statistical reliability of that component. The full-protocol comparison does not show a statistically detectable difference between the single and dual branches (full minus single branch: Δκ=−0.0013, 95% CI: −0.0104 to 0.0077; Holm-adjusted p=0.846), so a benefit from the extra CNN branch is not established. The five-channel model does outperform matched C4-only input (full minus C4-only: Δκ=0.0333, 95% CI: 0.0098–0.0569; Holm-adjusted p=0.0078), supporting complementary information from the additional signals within this pipeline. This does not imply statistical superiority over Bose et al.’s unmatched single-channel protocol.

In the single-fold pilot, removing self-attention changes κ by −0.018, while removing feature gating changes it by only −0.001. Neither estimate has replication across folds, so the study does not establish a performance benefit for these modules. Feature gating is retained as part of the prespecified compact architecture with 33,088 parameters of overhead; its utility should be tested independently. The average attention profile in Figure 11 is compatible with center-weighted local context but is not a statistical explanation of the prediction process.

Fold 0 occlusion ranks the channels C4 > EOG1 > C3 > EOG2 > EMG. This ordering is compatible with the central role of EEG in AASM staging and the contribution of eye movements to Wake, N1, and REM. However, occlusion measures sensitivity in one trained model and can reflect redundancy among correlated channels; it does not measure the independent physiological importance of each signal.

The representative Grad-CAM maps show broader and more localized saliency patterns in some slow- and fast-branch examples, respectively. This is consistent with the intended receptive field difference, but specific spindles, K-complexes, or eye movements are not independently marked in the displayed epochs. The maps therefore generate a physiological interpretation to test, rather than proving clinician-equivalent reasoning.

The ISRUC evaluation changes population (Indian stroke patients to Portuguese healthy subjects), site and equipment, sampling rate, montage convention, and class distribution simultaneously. The ensemble obtains κ=0.614 without ISRUC fine-tuning, a numerical difference of −0.020 from the iSLEEPS cross-validation mean. This comparison is not paired and does not isolate the source of domain shift. ISRUC-trained 3DSleepNet and MixSleepNet report κ=0.783 and 0.782, respectively, providing an in-domain reference for the lower zero-shot result.

N3 and REM F1-scores are higher on ISRUC (0.785 and 0.794) than on iSLEEPS (0.698 and 0.649), whereas Wake recall falls from 83.2% to 54.3%. Differences in health status, class distribution, hardware, reference montage, and scoring can all contribute. The results show nontrivial transfer to this cohort, but they do not establish that the learned features are population-invariant.

Probability averaging raises κ from the mean individual-model value of 0.580 to 0.614. No paired significance test is performed for this post hoc ensemble comparison, and two constituent models have higher point estimates. Deployment across clinical environments therefore remains to be evaluated prospectively.

The source resource contains 100 acute stroke recordings, of which 95 meet the common five-channel extraction criteria. This remains a single-country, single-clinical-population study. Weighted loss and label smoothing address class imbalance during training, but N1 and REM performance remains lower than that of the prevalent classes. Multi-site data, pretraining, focal loss [35], or alternative sampling strategies may improve minority-stage performance and should be compared under the same subject-level protocol.

Only one external dataset, comprising 10 healthy Portuguese adults, is evaluated. Generalizability therefore remains uncertain across pediatric and older age groups, other sleep and neurological disorders, additional geographic populations, scoring teams, acquisition protocols, reference montages, and recording hardware. Evaluation on datasets such as MASS and SHHS and on cohorts with narcolepsy, insomnia, or Parkinson’s disease would test these axes separately. The inference benchmark demonstrates speed and memory feasibility on the tested CPU/GPU, but no prospective reader study, workflow integration, calibration analysis, or clinical outcome evaluation was performed. Accordingly, the model is a research reference rather than a clinically validated system.

Future efforts will focus on (1) transfer learning from large-scale sleep datasets; (2) advanced class-imbalance techniques including focal loss and dynamic oversampling; (3) incorporation of additional PSG channels (occipital EEG, respiratory signals); (4) multi-task learning for joint sleep staging and OSA severity classification; (5) domain adaptation strategies to further improve cross-dataset performance; and (6) evaluation across diverse clinical populations to broaden the model’s applicability.

## 6. Conclusions

This study described SleepStageNet, a compact 1.075M-parameter model that integrates dual-branch convolutional encoding, feature gating, a bidirectional GRU, and multi-head self-attention for five-class staging from five PSG channels.

Of the 100 iSLEEPS source recordings, 95 met the common five-channel extraction criteria. Subject-independent 10-fold cross-validation on 78,323 epochs produced 73.91% accuracy (95% CI: 72.31–75.51%), a 67.29% macro F1-score (95% CI: 65.89–68.70%), and κ=0.634 (95% CI: 0.613–0.654). A matched single branch obtained κ=0.635 without evidence of a dual-branch advantage, whereas matched C4-only input obtained κ=0.600 and was lower than the five-channel model by 0.0333 (Holm-adjusted p=0.0078). The separate single-fold component screen remains exploratory, and the explanation analyses characterize model sensitivity without establishing a physiological mechanism.

Without ISRUC training or fine-tuning, the 10-model ensemble obtained κ=0.614 on ISRUC-SLEEP Subgroup III (10 healthy subjects; 8889 epochs). This result demonstrates nontrivial transfer to one external cohort, but broader generalization across populations, disorders, sites, montages, scoring protocols, and hardware requires additional evaluation.

The evaluated model, matched comparisons, computational benchmark, and zero-shot test provide a reproducible reference for further work on iSLEEPS and neurological cohorts. Prospective clinical evaluation and broader external validation are necessary before deployment.

## Figures and Tables

**Table 1:** Summary statistics of the iSLEEPS source cohort and the analyzed set.

Characteristic	Value
Number of subjects in source cohort	100
Number of subjects in analyzed set	95
Sex (male/female)	77/23
Age (years)	50.5±12.0 (22–78)
BMI (kg/m^2^)	28.0±13.1 (15.1–106.0)
AHI (events/hour)	25.2±20.5 (0.0–81.6)
Recording duration	∼6–8 h
Native sampling rate	256 Hz
Number of channels used	5 (C3, C4, EOG1, EOG2, EMG)
Epoch duration	30 s
Total annotated epochs	78,323

**Table 2:** Class distribution across the iSLEEPS dataset.

Stage	Epochs	Proportion (%)	Duration (h)
Wake (W)	23,812	30.40	198.4
N1	8739	11.16	72.8
N2	33,046	42.19	275.4
N3	6733	8.60	56.1
REM	5993	7.65	49.9
Total	78,323	100.00	652.7

**Table 3:** Class distribution of ISRUC-SLEEP Subgroup III.

Stage	Epochs	Proportion (%)
Wake (W)	1817	20.4
N1	1248	14.0
N2	2678	30.1
N3	2035	22.9
REM	1111	12.5
Total	8889	100.0

**Table 4:** Detailed architecture of SleepStageNet.

Component	Layer Details	Output
Input	PSG epoch	5×3840
	Epoch sequence	21×5×3840
Slow Branch	Conv1d (5, 64, k = 50, s = 6, p = 22)	64×640
	BN → GELU → MaxPool (8)	64×80
	Dropout (0.15)	
	Conv1d (64, 128, k = 8, p = 3)	128×79
	BN → GELU → MaxPool (4) → AdaptiveAvgPool (1)	128×1
Fast Branch	Conv1d (5, 64, k = 8, s = 2, p = 3)	64×1920
	BN → GELU → MaxPool (4)	64×480
	Dropout (0.15)	
	Conv1d (64, 128, k = 4, p = 1)	128×479
	BN → GELU → MaxPool (4) → AdaptiveAvgPool (1)	128×1
Feature Gating	FC (256, 64) → GELU	64
	FC (64, 256) → Sigmoid	256
Projection	Linear (256, 256) → GELU → Dropout (0.3)	256
BiGRU	2-layer BiGRU (256, 128)	21×256
	Dropout = 0.3, bidirectional	
Self-Attn	MultiHead (d = 256, heads = 4)	21×256
	Residual + LayerNorm; extract center	256
Classifier	Dropout (0.5)	256
	Linear (256, 5) (logits)	5
Total Trainable Parameters		1,075,269

**Table 5:** Overall 10-fold cross-validation results on iSLEEPS.

Fold	Accuracy (%)	Macro F1 (%)	Cohen’s κ
0	73.80	66.85	0.632
1	74.50	67.15	0.642
2	72.37	68.35	0.616
3	71.23	64.11	0.599
4	75.61	69.14	0.660
5	77.52	71.22	0.682
6	76.21	66.82	0.654
7	73.15	66.13	0.619
8	74.44	67.52	0.645
9	70.29	65.64	0.589
Mean ± Std	73.91±2.24	67.29±1.96	0.634±0.029

**Table 6:** Per-class performance (Mean ± Std across 10 folds).

Stage	Precision	Recall	F1-Score	Support
Wake	0.823±0.032	0.832±0.039	0.822±0.029	23,812
N1	0.367±0.044	0.439±0.063	0.395±0.048	8739
N2	0.797±0.029	0.810±0.043	0.801±0.033	33,046
N3	0.694±0.088	0.729±0.096	0.698±0.063	6733
REM	0.681±0.063	0.639±0.058	0.649±0.043	5993

**Table 7:** Model size, inference latency, throughput, and memory on the tested hardware.

Quantity	Setting	Measured Value
Trainable parameters	SleepStageNet	1,075,269
Serialized state dictionary	FP32	4.12 MiB
CPU latency per sequence	1 thread, batch 1	78.33 ms
CPU latency per sequence	8 threads, batch 1	20.63 ms
CPU process resident memory	8 threads, batch 1	547.9 MiB
GPU latency per sequence	batch 1	4.04 ms
GPU peak allocated/reserved memory	batch 1	48.95/58.0 MiB
GPU batch latency and throughput	batch 128	36.63 ms; 3494 sequences/s
GPU peak allocated/reserved memory	batch 128	1627/2002 MiB

**Table 8:** Exploratory component screen (validation Fold 0; maximum 30 epochs).

Variant	Acc (%)	F1 (%)	κ	Δκ	Params
Full Model	73.78	67.08	0.630	0.000	1075 K
No Self-Attention	72.35	65.53	0.612	−0.018	812 K
No Feature Gating	73.46	66.58	0.629	−0.001	1042 K
Single Branch	74.39	68.53	0.638	+0.007	974 K
No Augmentation	73.40	66.82	0.627	−0.004	1075 K
Seq Length = 11	73.53	66.58	0.628	−0.003	1075 K
Seq Length = 31	73.20	67.46	0.625	−0.006	1075 K
No BiGRU	65.97	60.76	0.542	−0.089	219 K

**Table 9:** Full-protocol matched 10-fold comparisons. Values are mean ± sample standard deviation; parentheses give the two-sided 95% confidence interval across folds.

Configuration	Channels	Parameters	Accuracy (%)	Macro F1 (%)	Cohen’s κ
Full, dual branch	5	1,075,269	73.91±2.24 (72.31–75.51)	67.29±1.96 (65.89–68.70)	0.634±0.029 (0.613–0.654)
Single branch	5	973,509	73.94±2.38 (72.24–75.65)	67.55±2.22 (65.97–69.14)	0.635±0.030 (0.614–0.657)
Full, C4 only	1	1,060,421	71.55±2.85 (69.51–73.59)	63.90±3.00 (61.75–66.05)	0.600±0.037 (0.574–0.627)

**Table 10:** Prespecified paired comparisons of fold-level Cohen’s κ. Differences are full model minus variant; positive values favor the full model.

Comparison	Mean Difference (95% CI)	*W*	Raw *p*	Holm *p*	$r_{\mathrm{rb}}$
Full vs. single branch	−0.0013 (−0.0104,0.0077)	25	0.846	0.846	−0.091
Full vs. C4 only	0.0333 (0.0098,0.0569)	1	0.0039	0.0078	0.964

**Table 11:** Channel importance via occlusion analysis.

Channel	κ-Drop	Relative Importance (%)
C4 (Right Central EEG)	0.096	40.8
EOG1 (Left Eye)	0.044	18.9
C3 (Left Central EEG)	0.034	14.4
EOG2 (Right Eye)	0.033	14.1
EMG (Chin)	0.027	11.7
Base κ (All Channels)	0.6217	

**Table 12:** Per-fold model performance on ISRUC-SLEEP Subgroup III.

Fold	Accuracy (%)	Macro F1 (%)	Cohen’s κ
0	69.46	66.40	0.607
1	65.73	64.36	0.553
2	65.58	62.87	0.563
3	66.28	64.10	0.566
4	72.16	69.47	0.639
5	66.50	63.29	0.568
6	71.84	70.07	0.635
7	65.76	63.03	0.558
8	66.26	64.33	0.562
9	64.32	59.89	0.546
Mean ± Std	67.39±2.76	64.78±3.14	0.580±0.033

**Table 13:** Reference points for ISRUC-SLEEP Subgroup III. Published models were trained on ISRUC-S3; SleepStageNet was trained only on iSLEEPS and evaluated zero-shot.

Method	ISRUC Training	Accuracy (%)	Macro F1 (%)	κ
3DSleepNet [33]	Yes, 10-fold CV	83.2	81.4	0.783
MixSleepNet [34]	Yes, 10-fold CV	83.0	82.1	0.782
SleepStageNet ensemble	No (zero-shot)	70.14	67.65	0.614

**Table 14:** Per-class ensemble performance on ISRUC-SLEEP Subgroup III.

Stage	Precision	Recall	F1-Score
Wake	0.931	0.543	0.686
N1	0.398	0.352	0.374
N2	0.646	0.878	0.744
N3	0.934	0.677	0.785
REM	0.670	0.972	0.794

**Table 15:** Per-subject ensemble performance on ISRUC-SLEEP Subgroup III.

Subject	Accuracy (%)	Macro F1 (%)	Cohen’s κ
1	70.96	64.51	0.602
2	72.90	67.36	0.641
3	72.33	66.03	0.620
4	59.32	55.74	0.485
5	72.25	65.47	0.629
6	72.45	65.83	0.625
7	60.44	59.88	0.511
8	76.70	70.58	0.695
9	71.52	65.37	0.605
10	69.47	72.55	0.614
Mean ± Std	69.83±5.47	65.33±4.54	0.603±0.059

## Data Availability

The iSLEEPS dataset is publicly available via Zenodo (https://doi.org/10.5281/zenodo.14873844, accessed on 4 August 2026) and the Data Foundation/IHub-Data portal (https://india-data.org/dataset-details/0b801dfa-4e42-4ec6-9c56-c6892b907ed2, accessed on 4 August 2026). The ISRUC-SLEEP dataset is publicly available at https://sleeptight.isr.uc.pt/ (accessed on 4 August 2026). The code for SleepStageNet will be made publicly available upon publication of the manuscript.

## Abbreviations

The following abbreviations are used in this manuscript:

PSG	Polysomnography
EEG	Electroencephalography
EOG	Electrooculography
EMG	Electromyography
AASM	American Academy of Sleep Medicine
CNN	Convolutional Neural Network
GRU	Gated Recurrent Unit
SE	Squeeze-and-Excitation
REM	Rapid Eye Movement
OSA	Obstructive Sleep Apnea
AHI	Apnea–Hypopnea Index
